# Intestinal Epithelial Cell Ferroptosis in Ulcerative Colitis: Pathogenesis, Signaling Networks, and Therapeutic Implications

**DOI:** 10.1007/s11596-026-00201-z

**Published:** 2026-05-19

**Authors:** Zhi-qiang Zhao, Zhou-xin Yu, Zhi-qiu Liu, Ting Yu, Heng Fan

**Affiliations:** Union Hospital, Tongji Medical College, Huazhong University of Science and Technology, Wuhan, 430022 China

**Keywords:** Ferroptosis, Ulcerative colitis, Intestinal epithelial cells, Therapeutic strategies, Lipid peroxidation, Iron overload, Antioxidant system dysregulation

## Abstract

The ferroptosis of intestinal epithelial cells (IECs), an iron-dependent form of cell death driven by lipid peroxidation, has emerged as a critical pathogenic driver of ulcerative colitis (UC). This review summarizes the core hallmarks of IEC ferroptosis in UC—specifically, lipid peroxidation, iron overload, and antioxidant system dysregulation—and describes key regulatory signaling networks, including the Nrf2/HO-1, SLC7A11/GPX4, and AMPK/mTOR pathways. Furthermore, we systematically evaluated emerging therapeutic strategies targeting these mechanisms, categorized into antioxidant activation, iron and lipid metabolism regulation, immune and microbiota modulation, and multitarget interventions. Elucidating this complex ferroptotic regulatory network provides a vital theoretical foundation for the development of novel disease-stage-specific therapeutic paradigms for UC management.

## Introduction

Inflammatory bowel disease (IBD), which primarily encompasses ulcerative colitis (UC) and Crohn’s disease (CD) [1], is a chronic, refractory disorder associated with substantial morbidity and significantly impaired health-related quality of life (HRQoL), despite its low overall mortality [2]. UC is specifically characterized by continuous mucosal inflammation that typically originates in the rectum and extends proximally throughout the colon. Epidemiological data indicate a rising global incidence of UC, potentially linked to advancing socioeconomic status and lifestyle modifications. The cardinal clinical manifestations include bloody diarrhea, abdominal pain, and unintended weight loss [3]. Conventional pharmacological therapies for UC currently rely on 5-aminosalicylates (5-ASAs), corticosteroids, and immunomodulators (e.g., thiopurines) [4]. However, the clinical utility of these agents is limited by suboptimal long-term efficacy, high relapse rates, considerable financial burdens, and significant adverse effects. For instance, prolonged sulfasalazine therapy may induce oxidative stress, hematologic abnormalities, and infertility [5], whereas chronic corticosteroid use is associated with severe metabolic complications. Consequently, novel clinical strategies with improved efficacy and safety profiles for managing UC are needed.

Although the specific etiology and pathogenesis of UC remain incompletely understood, accumulating evidence indicates that its development involves a multifactorial interplay of genetic susceptibility, immune dysregulation, mucosal barrier disruption, dysbiosis, and environmental factors [6, 7]. Critically, chronic and excessive intestinal inflammation constitutes a core pathogenic driver. This inflammation induces the apoptosis and necrosis of intestinal epithelial cells (IECs), further compromising mucosal barrier integrity. Consequently, mucosal damage and inflammation form a vicious cycle that drives disease progression [8]. The intestine, which serves as a primary site for immune defense and endocrine signaling, is particularly vulnerable to reactive oxygen species (ROS)-induced damage because of its high metabolic activity, which involves continuous biotransformation and energy production. Accordingly, studies have demonstrated significantly elevated ROS levels in the intestinal mucosa of UC murine models compared with those in healthy controls [9, 10]. Under physiological conditions, intestinal macrophages generate ROS as a key antimicrobial defense mechanism to eliminate phagocytosed pathogens [11]. However, in UC, pathologically elevated ROS attack polyunsaturated fatty acids (PUFAs) within IECs, initiating lipid peroxidation cascades that generate abundant lipid hydroperoxides (LOOHs). This oxidative damage serves as a principal trigger for IEC ferroptosis. Ferroptosis severely disrupts intestinal epithelial barrier function, thereby accelerating the pathogenesis of UC [12].

**Fig. 1:**
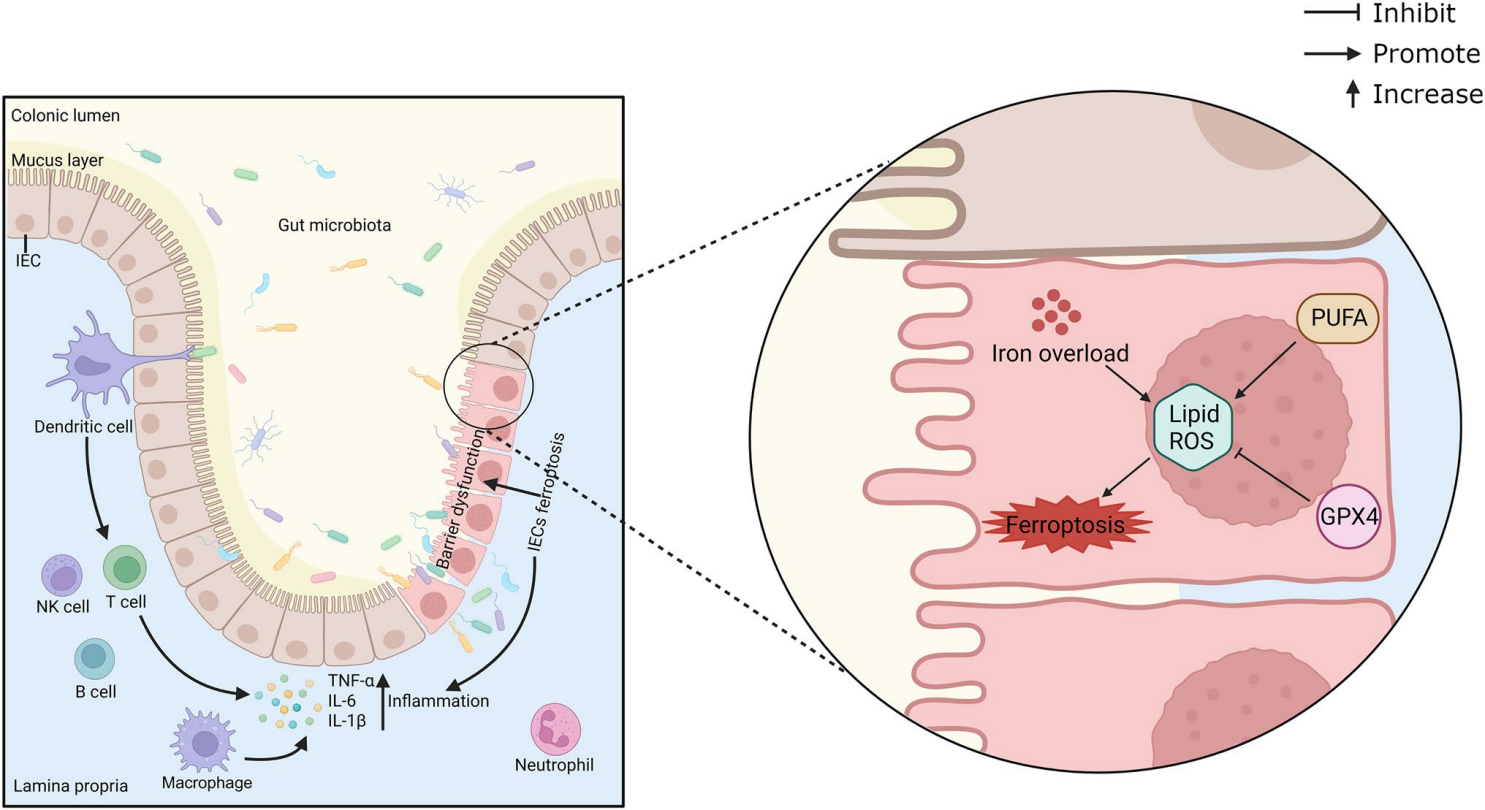
IEC ferroptosis is a critical pathogenic driver in UC. Current evidence demonstrates that IEC ferroptosis directly drives UC pathogenesis by disrupting the intestinal mucosal barrier and triggering immune cell hyperactivation. This cascade subsequently elicits the excessive production of pro-inflammatory cytokines, ultimately culminating in chronic intestinal inflammation and profound epithelial damage (GPX4 precisely scavenges lipid peroxides to inhibit ferroptosis)

Recent evidence has established a pathogenic link between the ferroptosis of IECs and the progression of UC [13, 14]. Ferroptosis is an iron-dependent form of regulated cell death driven by lipid peroxidation. It is characterized by intracellular iron overload, glutathione (GSH) depletion, the inactivation of glutathione peroxidase 4 (GPX4), and the lethal accumulation of lipid peroxides [15]. During the initiation of ferroptosis, redox-active iron catalyzes excessive ROS generation via the Fenton reaction, which propagates the peroxidation of PUFAs. This process directly compromises plasma membrane integrity, ultimately culminating in lytic cell death. Furthermore, ferroptosis has been implicated in diverse pathological conditions, including malignancies, cardiovascular disorders, neurodegenerative diseases, ischemia‒reperfusion injury, and UC [16–19]. Experimental studies have demonstrated that diminished GSH levels and suppressed GPX4 activity in the IECs of UC model mice impair the detoxification of lipid peroxides, thereby exacerbating IEC ferroptosis [17]. Consequently, the mechanistic role of IEC ferroptosis in UC pathogenesis warrants further elucidation, as therapeutic targeting of this pathway represents a highly promising strategy for clinical intervention (Fig. 1).

## Core Hallmarks of Ferroptosis and Their Pathophysiological Relevance to UC

### Lipid Peroxides Drive the Pathogenesis and Progression of UC

Fatty acids are essential bioactive mediators that serve as fundamental structural components of biological membranes and participate in critical physiological functions, including the regulation of energy homeostasis, endogenous hormone biosynthesis [20], anti-inflammatory responses [21], and cardiovascular maintenance [22]. On the basis of their degree of carbon chain unsaturation, fatty acids are classified into saturated fatty acids (SFAs), monounsaturated fatty acids (MUFAs), PUFAs, and trans fatty acids. Crucially, PUFAs play a specific regulatory role in ferroptosis-associated lipid peroxidation. The enzyme acyl-CoA synthetase long-chain family member 4 (ACSL4, a lipid metabolic enzyme that provides the essential substrates for ferroptosis) catalyzes the ATP-dependent esterification of long-chain fatty acids (including ω-3 and ω-6 PUFAs) to generate PUFA-acyl-CoAs. These intermediates are subsequently incorporated into membrane phospholipids as PUFA-phospholipids (PUFA-PLs) via lysophosphatidylcholine acyltransferase 3 (LPCAT3, a key mediator of phospholipid remodeling)-mediated reacylation [23]. During the induction of ferroptosis, ROS propagate peroxidative damage to these PUFA-PLs, which disrupts membrane fluidity and integrity, ultimately causing plasma membrane rupture [12]. Notably, colonization by adherent-invasive *Escherichia coli* (AIEC) combined with arachidonic acid (AA) supplementation exacerbates colitis in dextran sulfate sodium (DSS)-treated mice; this effect is abolished by the ferroptosis inhibitor ferrostatin-1 (Fer-1) [24]. Given the pivotal role of ACSL4 in lipid metabolism and ferroptosis, substantial evidence has demonstrated that promoting ACSL4 ubiquitination, phosphorylation, or proteasomal degradation confers significant cellular resistance to ferroptotic cell death [25–27]. Furthermore, arachidonate 5-lipoxygenase (ALOX5, the primary rate-limiting enzyme in leukotriene synthesis), an iron-containing non-heme dioxygenase, catalyzes the 5-lipoxygenation of AA to generate bioactive lipid mediators—such as leukotrienes (LTs), lipoxins (LXs), and 5-hydroxyeicosatetraenoic acid (5-HETE)—which serve as key regulators of inflammatory cascades [28]. Experimental studies have indicated that ALOX5 inhibition significantly attenuates lipid peroxidation, preserves mitochondrial ultrastructure, suppresses inflammatory responses, and ameliorates DSS-induced colitis without substantially altering GPX4 activity or ferric ion concentrations [29].

### Potential Role of Iron Overload in UC

Iron, an essential trace element, exists primarily as ferrous (Fe^2+^) and ferric (Fe^3+^) ions within hemoglobin and ferritin complexes. It plays critical physiological roles in oxygen transport, cellular respiration, energy metabolism, DNA biosynthesis, and immune regulation [30]. Ferritin, which is composed of heavy (FTH1) and light (FTL) chain subunits, sequesters excess intracellular Fe^3+^ in a redox-inert form. This storage mechanism mitigates Fenton reaction-mediated ROS generation by limiting the availability of the labile iron pool [31]. Nuclear receptor coactivator 4 (NCOA4) functions as a selective autophagy receptor for ferritinophagy, mediating the translocation of ferritin to lysosomes for degradation [32]. The upregulation of NCOA4 accelerates ferritinophagy, thereby releasing labile Fe^2+^, which potentiates ROS generation and propagates lipid peroxidation, ultimately inducing ferroptosis [33–35]. Hepcidin, a hepatocyte-derived peptide hormone, serves as the principal regulator of systemic iron homeostasis [36]. It binds to ferroportin (FPN1)—the sole cellular iron exporter—inducing its internalization and degradation to inhibit iron efflux and reduce plasma iron concentrations [37]. However, during inflammation, pro-inflammatory cytokines stimulate hepcidin overexpression, which subsequently suppresses FPN1 expression and promotes iron retention within hepatocytes and macrophages. This sequestration expands intracellular labile iron pools, heightening cellular susceptibility to ferroptosis [38]. Heme oxygenase-1 (HO-1), a typically cytoprotective enzyme, catalyzes heme degradation to generate biliverdin (which is subsequently converted to bilirubin), carbon monoxide (CO), and free Fe^2+^. The released iron undergoes cytosolic redistribution via iron chaperones (e.g., PCBP1/2) for reutilization, or it is directed to storage proteins (e.g., ferritin) for sequestration. Paradoxically, sustained HO-1 activation can exceed the cellular iron-buffering capacity, leading to a labile iron overload that overwhelms ferritin storage mechanisms and potentiates ferroptosis [39]—an effect that can be rescued by the genetic ablation or silencing of HO-1 [40, 41].

### Dysregulation of the Antioxidant System Exacerbates UC Pathogenesis

Programmed cell death arises from a disrupted equilibrium between prodeath signaling and endogenous cytoprotective mechanisms. A pivotal event in the pathogenesis of ferroptosis is the disruption of antioxidant homeostasis. As the core molecular hub of the endogenous antioxidant defense system, the GSH‒GPX4 regulatory axis plays a critical role in maintaining cellular redox homeostasis and inhibiting ferroptosis [42]. GPX4, a selenium-dependent oxidoreductase within the GPX superfamily [43], exists as three functionally distinct isoforms with compartment-specific localizations: mitochondrial (mGPX4), nuclear (nGPX4), and cytosolic (cGPX4) [44]. As the sole enzyme capable of directly reducing membrane-integrated LOOHs, GPX4 constitutes the primary defense against ferroptosis by preventing iron-catalyzed lipid peroxidation cascades within phospholipid bilayers [45, 46]. Consequently, genetic or pharmacological ablation of GPX4 robustly induces ferroptosis and suppresses cellular proliferation [47, 48]. For instance, the small molecule RSL3 selectively inhibits GPX4 through covalent modification of its catalytic selenocysteine (Sec46) and adjacent cysteine (Cys66) residues, thereby inactivating its lipid peroxide reductase activity. This inactivation leads to the lethal accumulation of lipid peroxides and the subsequent execution of ferroptosis [49]. Moreover, GSH, the predominant endogenous antioxidant in mammalian cells, is involved in essential physiological processes, including redox buffering, xenobiotic detoxification, and signaling modulation [50]. As a tripeptide comprising glutamate, cysteine, and glycine, GSH utilizes its reactive thiol group (–SH) to directly scavenge ROS and lipid peroxides, thereby preserving cellular integrity against oxidative damage [51]. The GSH/GSSG redox couple is dynamically maintained through GPX-mediated oxidation and glutathione reductase (GSR)-dependent reduction [50, 52]. Compared with that in healthy controls, the expression of the γ-glutamylcysteine ligase catalytic subunit (GCLC), the rate-limiting enzyme in GSH biosynthesis, in the colonic mucosa in patients with UC is significantly lower, resulting in impaired GSH synthesis [53]. Consequently, the pharmacological restoration of GSH levels effectively mitigated ferroptosis in experimental UC models [54, 55].

## Core Signaling Pathways Modulating IECs Ferroptosis in UC Pathogenesis

### Nrf2/HO-1 Pathway

**Fig. 2:**
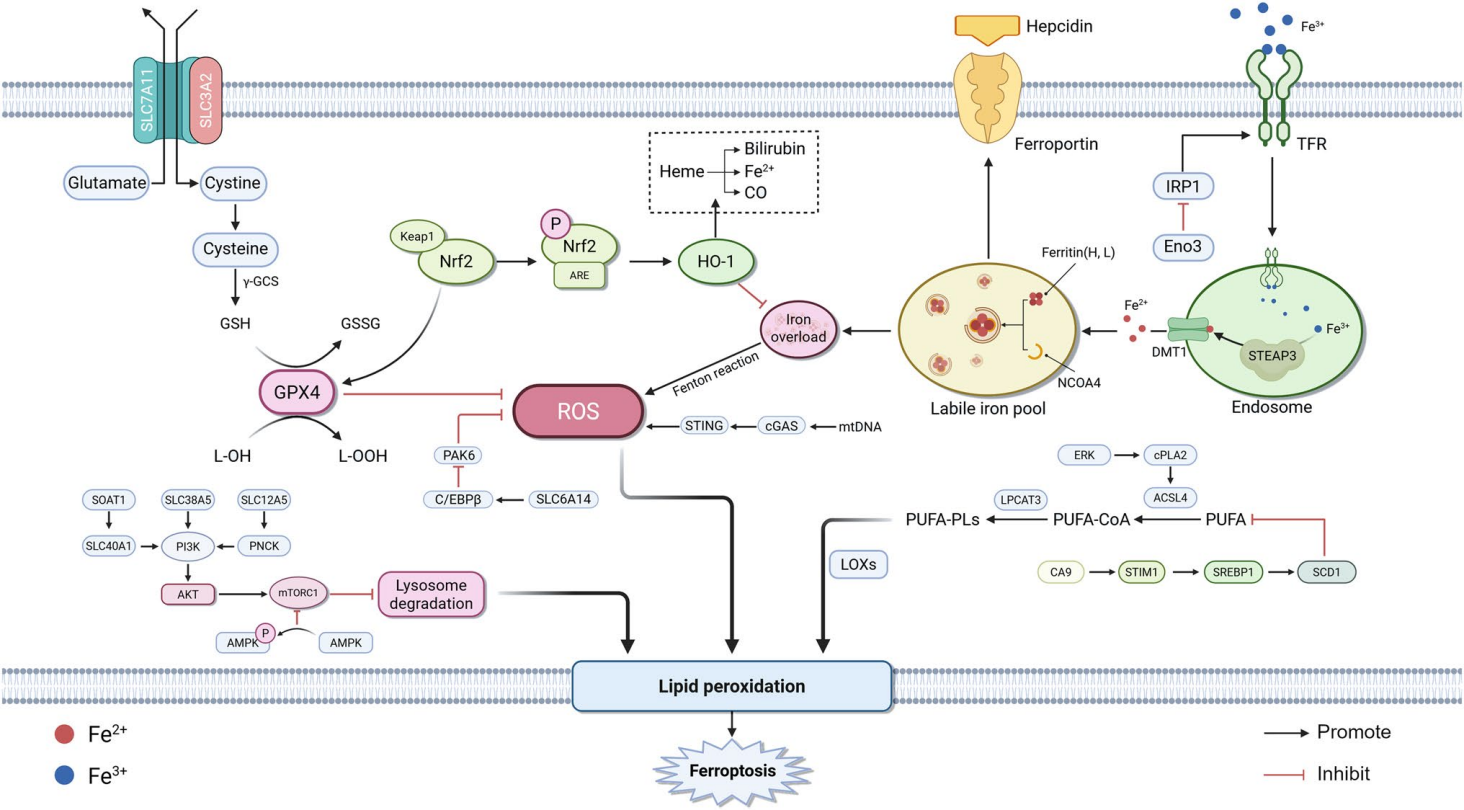
The molecular mechanisms and principal regulatory pathways governing ferroptosis in IECs involve three core metabolic axes: the amino acid/GSH pathway, the lipid peroxidation cascade, and the iron homeostasis network. These processes are coordinately regulated through integrated signaling circuits and transcriptional regulators. The key molecular determinants and signaling networks modulating IEC ferroptosis in UC pathogenesis: ACSL4, lipid metabolic enzymes that provide the “fuel” for ferroptosis; AKT, the central integrator of cellular signaling networks; AMPK, the master regulator of cellular energy homeostasis; GSH, the ubiquitous intracellular reductant; GPX4, precisely scavenging lipid peroxides to inhibit ferroptosis; HO-1, the rate-limiting enzyme in heme metabolism; mTORC1, a sophisticated activation mechanism that integrates convergent inputs from the AKT and AMPK signaling pathways; Nrf2, the master regulator of the cellular antioxidant response; PI3K, the master regulator of cellular growth signaling

Nuclear factor erythroid 2-related factor 2 (Nrf2) is a master transcriptional regulator of the cellular antioxidant response. Under basal conditions, Kelch-like ECH-associated protein 1 (KEAP1) targets Nrf2 for ubiquitin-proteasomal degradation, maintaining its low constitutive intracellular levels [56]. Upon exposure to oxidative stress, ROS or electrophilic stressors modify specific cysteine residues within KEAP1, thereby disrupting the KEAP1–Nrf2 complex. This dissociation enables Nrf2 stabilization, nuclear translocation, and subsequent binding to antioxidant response elements (AREs), which initiates the transcription of downstream cytoprotective genes (e.g., *HMOX1*, *NQO1*, *GCLC*, and *GCLM*). Consequently, Nrf2 activation enhances GSH biosynthesis, ROS detoxification, and mitochondrial biogenesis via the upregulation of NRF1 and PPARGC1A expression [57, 58]. HO-1—encoded by the key Nrf2 target gene *HMOX1*—is the inducible isoform of heme oxygenase that catalyzes the degradation of heme into biliverdin [59]. Biliverdin is subsequently reduced to the potent antioxidant bilirubin; together, these molecules effectively scavenge ROS and attenuate oxidative stress. Furthermore, the concomitantly released labile iron is sequestered by ferritin, which mitigates iron overload-induced lipid peroxidation and subsequent ferroptosis [58]. Critically, the colonic mucosa of UC model mice exhibited suppressed Nrf2/HO-1 signaling, concomitant with reduced GPX4 activity and elevated levels of lipid peroxidation markers (e.g., malondialdehyde and 4-HNE). Pharmacological activation of Nrf2 effectively attenuates DSS-induced colitis and inhibits IEC ferroptosis (Fig. 2) [60–62]. Furthermore, the modulation of the Nrf2/HO-1 pathway upregulates the expression of tight junction proteins (e.g., ZO-1 and occludin), preserves intestinal epithelial barrier integrity, and ameliorates intestinal hyperpermeability [63–65].

### SLC7A11/GPX4 Pathway

Solute carrier family 7 member 11 (SLC7A11), the light-chain subunit of the system xc⁻ antiporter, forms a functional heterodimer with the heavy-chain subunit SLC3A2 [66]. This complex mediates cystine/glutamate exchange at a 1:1 stoichiometry, exporting intracellular glutamate while importing extracellular cystine. Upon cellular entry, cystine is rapidly reduced to cysteine, serving as the rate-limiting precursor for GSH biosynthesis—a process essential for maintaining redox homeostasis and suppressing ferroptosis [67]. GPX4, a selenocysteine-containing antioxidant enzyme, utilizes GSH as a reducing cofactor to catalyze the reduction of LOOHs into nonreactive lipid alcohols. This catalytic activity preserves membrane integrity by preventing the oxidative degradation of phospholipid bilayers [42–44]. The functional interdependence of these molecules results in the formation of the canonical SLC7A11/GSH/GPX4 signaling axis. Disruption of this axis via SLC7A11 inhibition diminishes cystine uptake and GSH synthesis. The resulting substrate deficiency impaired GPX4 catalytic efficiency, leading to lethal LOOH accumulation and the execution of ferroptosis. Clinically and experimentally, studies have demonstrated a notable decrease in GSH levels in both rat and mouse models of DSS-induced UC [68–70]. Conversely, targeted GSH supplementation markedly alleviates intestinal inflammatory responses [71]. Furthermore, while GPX4 inactivation induces IEC ferroptosis and drives UC pathogenesis, increasing GPX4 expression successfully reverses this pathological process [72, 73]. Similarly, the upregulation of SLC7A11 expression significantly suppresses ferroptosis [55, 74, 75], whereas its downregulation exacerbates it [76–78]. Therefore, therapeutic activation of the SLC7A11/GSH/GPX4 pathway precisely modulates intracellular GSH synthesis, attenuates oxidative stress, inhibits IEC ferroptosis, and ultimately impedes UC progression (Fig. 2) [79–81].

### AMPK/mTOR Pathway

AMP-activated protein kinase (AMPK), a serine/threonine kinase heterotrimer composed of catalytic (α), scaffolding (β), and regulatory (γ) subunits [82], orchestrates cellular energy homeostasis by promoting ATP-generating pathways (e.g., fatty acid oxidation), exerting anti-inflammatory and antioxidant effects, and modulating autophagy in a context-dependent manner [83]. Mechanistic target of rapamycin (mTOR) serves as a master regulator of cellular anabolism and proliferation [84]. It assembles into two structurally and functionally distinct complexes—mTORC1 and mTORC2—which coordinately regulate diverse processes, including protein translation, transcriptional regulation, and autophagic flux [85]. As a central integrator of intracellular energy metabolism and growth, mTOR intrinsically modulates these fundamental processes [86] and interacts closely with AMPK signaling [87]. Through continuous energy status sensing, this network orchestrates the cellular anabolic–catabolic balance. Notably, AMPK functions as an energy-deficit sensor, whereas mTOR operates as a nutrient-sufficiency commander; their reciprocal antagonistic effects govern cellular proliferation and survival. Pathological mTOR hyperactivation suppresses autophagy, culminating in the accumulation of damaged organelles and misfolded proteins. This triggers oxidative stress and mitochondrial dysfunction, ultimately accelerating IEC death. Conversely, AMPK activation during energy stress maintains metabolic homeostasis through mTORC1 inhibition and increases autophagy, fatty acid β-oxidation, and immunomodulation (via macrophage polarization and T-cell differentiation), collectively attenuating intestinal inflammation. Consequently, AMPK-mediated mTOR suppression reduces mucosal inflammation and enhances barrier function, thereby inhibiting UC progression [86–89]. Notably, GPX3 upregulation confers resistance to autophagy-associated ferroptosis via AMPK/mTOR pathway modulation [90], whereas ALOX5 promotes ferroptosis through this same axis (Fig. 2) [91]. Furthermore, the mechanosensitive ion channel Piezo1 is overexpressed in patients with UC and in DSS-induced colitis models. Epithelial Piezo1 deficiency attenuates ferroptosis through AMPK/mTOR pathway activation, thereby reducing mucosal inflammation and enhancing barrier integrity [92].

### PI3K/AKT/mTOR (PAM) Pathway

The PAM pathway is a pivotal intracellular signaling network critically involved in regulating cellular growth, proliferation, survival, metabolic homeostasis, and autophagic flux [93]. Phosphatidylinositol 3-kinase (PI3K), a class I lipid kinase, comprises catalytic (e.g., p110α and p110β) and regulatory subunits (e.g., p85) [94]. Upon activation by receptor tyrosine kinases (RTKs) or G-protein-coupled receptors, PI3K catalyzes the generation of phosphatidylinositol-3,4,5-trisphosphate (PIP3) from phosphatidylinositol 4,5-bisphosphate (PIP2) at the plasma membrane. PIP3 subsequently serves as a secondary messenger to propagate downstream signals [95]. Protein kinase B (AKT)—the core effector of this pathway—undergoes membrane translocation via PIP3 binding, followed by dual phosphorylation at Thr308 by phosphoinositide-dependent kinase 1 (PDK1) and at Ser473 by mTORC2 to achieve full activation [96]. Pharmacological inhibition of this pathway attenuates colonic inflammation [97, 98], reduces oxidative and endoplasmic reticulum (ER) stress [99], improves mitochondrial function [100], and ultimately confers protection against UC. Paradoxically, while some studies have demonstrated that PI3K/AKT/mTOR inhibition suppresses ferroptosis by decreasing intracellular iron accumulation and ROS levels [101, 102], others have reported that pathway agonism inhibits ferroptosis [35, 103]. This discrepancy suggests that context-dependent regulation is influenced by cell type, microenvironmental factors, or inhibitor specificity. Furthermore, the SLC superfamily—the largest group of membrane transporters—exhibits functional crosstalk with this pathway. For instance, SLC12A5 upregulation induces ER stress, enhances calcium release, upregulates PNCK, and activates PI3K/AKT/mTOR signaling to inhibit ferroptosis while promoting tumorigenesis [104]; conversely, SLC38A5-mediated glutamine transport activates the PI3K/AKT/mTOR axis and enhances SREBP1/SCD-1 signaling to suppress ferroptosis [105]. Additionally, SOAT1 upregulates SLC40A1 to modulate ferroptosis sensitivity via PI3K/AKT/mTOR activation and regulation of intracellular iron levels (Fig. 2) [106].

The PI3K/AKT/mTOR pathway transduces growth signals to activate mTORC1, thereby inhibiting ferroptosis. Conversely, the AMPK pathway is activated by energy stress, leading to mTORC1 suppression, ferritinophagy induction, and ferroptosis promotion. The activation state of mTORC1 directly governs cellular sensitivity to ferroptosis: elevated mTORC1 activity confers resistance, whereas its inhibition sensitizes cells to this process. Furthermore, the interplay between these signaling cascades regulates the intracellular balance of pro-oxidant PUFAs and antioxidant MUFAs, ultimately dictating cellular susceptibility to ferroptotic cell death.

### Other Pathways

ACSL4, a pivotal lipid-metabolizing enzyme, drives ferroptosis by catalyzing the esterification of long-chain PUFAs—such as AA—into acyl-CoA derivatives. This process facilitates their incorporation into membrane phospholipids, thereby potentiating lipid peroxidation [107, 108]. Conversely, the downregulation of ACSL4 upregulates GPX4 expression, suppresses ferroptosis, and attenuates UC progression [81, 109, 110]. Notably, during UC pathogenesis, compared with M1 macrophages, M2 macrophages are more susceptible to ferroptosis, a phenomenon attributed to ERK/cPLA2/ACSL4-mediated AA metabolic activation [111]. Furthermore, protein interactions involving carbonic anhydrase IX (CA9, hypoxia-induced pH regulator) and stromal interaction molecule 1 (STIM1, a bidirectional transducer of calcium signaling) dissociate insulin-induced gene 2 (INSIG2) from the SREBP cleavage-activating protein (SCAP)–sterol regulatory element-binding protein 1 (SREBP1, the master transcriptional regulator of lipid synthesis) complex. This dissociation enables the Golgi translocation and proteolytic maturation of SREBP1, which subsequently transcriptionally activates stearoyl-CoA desaturase-1 (SCD1, the primary enzyme that catalyzes lipid desaturation). This signaling cascade enhances MUFA synthesis, reduces lipid peroxidation, and inhibits IEC ferroptosis in UC models (Fig. 2) [112].

In parallel, the cyclic GMP-AMP synthase–stimulator of interferon genes (cGAS–STING) pathway, a core mammalian innate immune signaling axis, causes the detection of aberrant cytoplasmic DNA (whether pathogen-derived or self-damaged) to initiate immune responses [113]. Recent evidence has indicated that mitochondrial DNA (mtDNA) release during cellular stress activates cGAS–STING signaling, triggering downstream inflammation and contributing to colitis pathology [114]. Additionally, SLC6A14 (the panamino acid “nutrient transporter”) is upregulated in UC and promotes IEC ferroptosis via the CCAAT/enhancer-binding protein β (C/EBPβ, a versatile transcription factor)–p21–activated kinase 6 (PAK6, a dual-function kinase bridging signal transduction and transcriptional regulation) axis [115]. In contrast, enolase 3 (ENO3, a muscle-specific glycolytic enzyme) upregulation mitigates ferroptosis in the colonic epithelium through the ENO3-iron regulatory protein 1 (IRP1, a central regulator of iron metabolism) axis, significantly ameliorating DSS-induced colitis (Fig. 2) [116].

## Crosstalk Between Ferroptosis and Other Forms of Cell Death in IECs of UC

In UC, ferroptosis in IECs does not occur in isolation; rather, it involves a complex interplay with apoptosis, pyroptosis, necroptosis, and autophagy-dependent cell death to form an intricate regulatory network [117]. This cross-talk is characterized primarily by four mechanisms: (1) ferroptosis and apoptosis share signaling molecules such as p53 and BID, with mitochondria serving as hubs that drive ferroptosis via ROS production while simultaneously initiating apoptosis through cytochrome c release [12]; (2) both ferroptosis and pyroptosis are triggered by excessive ROS and mitochondrial damage; as lytic cell death modes, these processes release significant quantities of damage-associated molecular patterns (DAMPs), thereby promoting a self-amplifying “death–inflammation” cascade [118]; (3) inflammatory stimuli such as TNF-α synergistically induce ferroptosis and necroptosis, which share downstream effectors, including lipid peroxidation and DAMP release [119]; (4) autophagy promotes ferroptosis through selective pathways—such as ferritinophagy and lipophagy—that degrade regulatory proteins (e.g., GPX4 and ferritin) to provide free iron and lipid substrates [120]. Moreover, the mTOR, AMPK, and p62/KEAP1/NRF2 axes constitute a bidirectional regulatory bridge between autophagy and ferroptosis [121]. Collectively, these interactions exacerbate intestinal barrier dysfunction and perpetuate inflammation, suggesting that therapeutic strategies targeting multiple programmed cell death pathways represent a promising direction for UC treatment.

## Therapeutic Strategies Targeting IEC Ferroptosis in UC Management

Therapeutic strategies targeting ferroptosis in UC are multifaceted and can be systematically classified into six distinct categories (Table 1).

**Table 1:** Therapeutic strategies targeting ferroptosis in UC

Regulatory axis	Specific strategy/representative agent	Key molecular/pathway targets	Core function/mechanism	Reference
Antioxidant core system	Selenium/seleno-amino acids, Se-HMPB Nanozym	Upregulate GPX4, activate Nrf2	Enhance GPX4 enzymatic activity; scavenge lipid peroxides	[122–125]
	Isorhamnetin, α-lipoic acid, curcumolide A	Activate Nrf2/HO-1 pathway	Induce downstream antioxidant enzyme expression; alleviate oxidative stress	[62, 149] [150]
	Hesperetin, Safflower Yellow, Pulsatilla decoction, Huang-Lian-Hou-Pu decoction, Gegen Qinlian decoction	Upregulate GPX4, SLC7A11; downregulate ACSL4; activate Nrf2	Synergistically enhance antioxidant capacity via multiple targets	[79, 110, 126, 127, 151]
	Vitamin D, magnolin	Upregulate GPX4; inhibit ALOX5	Reduce lipid peroxidation substrates; block the peroxidation chain	[29, 81]
	Glycyrrhizae decoction, An-Chang decoction	Activate Nrf2/HO-1; regulate p53/SLC7A11/GPX4	Reduce intracellular lipid peroxidation; inhibit ferroptosis	[134, 152]
	Berberine	Inhibit STAT1, activate Nrf2/SLC7A11/GPX4	Reduce STAT1-mediated suppression of Nrf2; enhance antioxidant capacity	[130]
	Heat shock protein family A member 5 (HSPA5)	Upregulate and activate GPX4	Enhance tight junctions; reduce ferroptosis and intestinal injury	[153]
	Ginsenoside Rh2	miR-125a-5p/SP1 axis	Regulate ferroptosis-related proteins via non-coding RNA	[154]
	Metformin, Piezo1 deletion	Activate AMPK; inhibit mTOR	Improve mitochondrial function; reduce ROS production	[92, 129]
	Ferrostatin-1, liproxstatin-1	Directly scavenge lipid peroxyl radicals	Inhibit ferroptosis and serve as positive controls	[62, 131, 132]
	Indigo/indirubin, Qing Dai	Upregulate Nrf2 downstream antioxidant genes	Increase GSH; resist lipid peroxidation	[13, 132]
	*Lespedeza bicolor* honey extract, Dandelion root polysaccharide	Activate Nrf2/HO-1; increase SOD, GSH	Comprehensively enhance antioxidant enzyme activity	[64, 65]
	Electroacupuncture	Activate Nrf2/HO-1; Upregulate GPX4, FTH1	Physical therapy; activate endogenous antioxidant systems	[128]
Iron metabolism regulation	Deferoxamine, deferasirox, deferiprone	Chelates labile iron (Fe^2+^)	Reduce Fenton reaction; decrease hydroxyl radical generation	[62, 70, 132]
	Isorhamnetin	Direct iron chelation	Dual mechanism: chelate iron and activate NRF2	[62]
	Liquiritin	Activate Prdx6; upregulate FTH1	Promote ferritin synthesis; increase iron storage	[133]
	Kumatakenin	Eno3–IRP1 axis	Modulate iron regulatory protein; reduce intracellular iron levels	[116]
Lipid metabolism reprogramming	Hesperetin, 6-gingerol	Inhibit ACSL4, PTGS2, ALOX5/15	Reduce peroxidation of PUFAs	[17, 110]
	Vitamin D, An-Chang decoction	Inhibit ACSL4	Specifically block ACSL4-mediated lipid peroxidation	[81, 134]
	Magnolol	Inhibit ALOX5	Block lipoxygenase pathway-driven lipid peroxidation	[29]
	Vanillic acid	Target CA9; activate SCD1	Regulate MUFA synthesis; counter ferroptosis	[112]
	Palmatine	Downregulate ACSL4, COX-2	Reduce lipid peroxidation and inflammation	[109]
Immune cell modulation	β-Caryophyllene	Activate CB2R in macrophages	Inhibit macrophage ferroptosis and subsequent inflammation	[135]
	Magnolol	Regulate M1/M2 macrophage polarization	Promote anti-inflammatory M2 phenotype; inhibit M1 phenotype	[29]
	Mineralized nano-inhibitor (CLF)	CaSR/AKT/β-catenin; promote M2 polarization	Immunomodulate and release Fer-1 to inhibit ferroptosis	[136]
	Se-HMPB Nanozyme	Inhibit T-cell differentiation	Modulate the intestinal immune barrier	[125]
	Mesenchymal stem cells (MSCs)	Upregulate MUC-1; modulate gut microbiota	Inhibit ferroptosis via multiple mechanisms; repair immune function	[155]
	ERC-derived exosomes, hucMSC-Ex	Downregulate ACSL4; upregulate GPX4	Delivers miRNAs; regulate immune and epithelial cells	[137, 138]
Gut microbiota modulation	Hesperetin, phlorizin, protocatechuic acid, sodium butyrate (NaB)	Modulate microbiota structure: increase probiotics (*Lactobacillus*, *Firmicutes*, *Prevotellaceae*), reduce pathobionts (*Proteobacteria*, *Erysipelotrichaceae*, *Clostridium*)	Indirectly inhibit ferroptosis via microbial metabolites (e.g., SCFAs)	[54, 110, 139, 140]
	Deferasirox	Remodel microbiota; increase SCFAs production	Dual mechanism: chelate iron and modulate microbiota	[70]
	*Tremella fuciformis* polysaccharide	Modulate microbiota composition; validated by FMT	Inhibit ferroptosis via microbiota dependence	[156]
	Probiotic microspheres (LGG@CT@CA)	Enhance probiotic colonization; reduce ROS and iron deposition	Live biotherapeutic; improve intestinal microenvironment	[157]
	Plasmalogen	Microbiota-derived ether lipid	Supplement key lipid metabolites; inhibit ferroptosis	[141]
	*Fusobacterium nucleatum*	Downregulate GPX4; upregulate FTH1/ACSL4; increase Fe^2+^/MDA	Disrupt intestinal epithelial barrier by inducing ferroptosis via pathogenic bacterium	[144]
Multi-target/integrated regulation	Pulsatilla decoction, Huang-Lian-Hou-Pu decoction, An-Chang decoction, Huang-Qin decoction, Kui-Jie-Ning acupoint application, Ge-Gen-Qin-Lian decoction, Li-Zhong decoction, Shao-Yao decoction	Multitarget: activate Nrf2/SLC7A11/GPX4, Keap1/Nrf2/HO-1; inhibit p53/ACSL4	Traditional Chinese medicine formulas or therapies; systematically regulate ferroptosis network	[79, 109, 126, 127, 134, 142, 143, 158]
	*Codonopsis pilosula* (Dangshen)	PI3K/Akt/Keap1/Nrf2 axis; regulate MIRO/DRP1	Suppress oxidative stress, inhibit ferroptosis, regulates mitochondrial dynamics	[159]
	Sodium butyrate (NaB)	Activate ERK/STAT3; modulate gut microbiota; regulates GPX4/SLC7A11/ACSL4	Dual mechanism: inhibits ferroptosis and remodels gut microbiota	[159]
	Xuejie San	FGL1/NF-κB/STAT3 loop, SLC7A11/GSH/GPX4	Integrate anti-inflammatory and antioxidant effects	[160]
	Oxymatrine	Regulate IL-1β, NOS2, HIF1A, DUOX2	Regulate multi-genes; reduce inflammation and ferroptosis	[14]
	Celecoxib	Upregulate GPX4, xCT; inhibit apoptosis	Target both ferroptosis and apoptosis	[161]
	MELK inhibitor (OTSSP167)	Inhibit AKT/IKK/P65 and ERK/IKK/P65 pathways	Suppress inflammatory and inhibit ferroptosis; impact tumorigenesis	[162]
	Neuropeptide substance P (SP)	cGAS–STING pathway	Regulate inflammation and ferroptosis via a novel mechanism	[114]
	PDE4 inhibitors (roflumilast, dipyridamole)	Inhibit PDE4; restore PKA/CREB/GPX4	Target VTN-induced ferroptosis; protect barrier function	[163]
	Gene overexpression: *NEDD4L*, *IGF2BP2*, *Furin*	Stabilize GPX4 protein or mRNA	Gene therapy strategies; enhance endogenous inhibitory mechanisms	[145, 146, 164]
	Gene silencing: *IRF7*, *LCN2*	Downregulate pro-ferroptotic genes	Block pathological ferroptotic signaling	[147, 148],
	Human breast milk-derived phospholipid (DOPE)	Regulate SLC7A11, GPX4, ACSL4	Nutritional supplement; inhibit multi-targets	[165]

### Antioxidant Core System Activation

This approach represents the most extensively investigated strategy in the field, primarily aiming to bolster the intrinsic antioxidant defense capacity of cells. The underlying mechanism involves the activation of the Nrf2/HO-1/GPX4 signaling axis, which upregulates key ferroptosis suppressors, including GPX4 and SLC7A11, in addition to other downstream antioxidant enzymes. Representative interventions include selenium supplementation [122–125]; the use of various natural flavonoids (e.g., hesperetin [110] and isorhamnetin [62]); traditional Chinese medicine (TCM) formulas (e.g., Pulsatillae, Coptidis [126] and *Magnoliae officinalis* [127]; and Puerariae, Scutellariae and *Coptidis decoctions* [79]); and physical therapies such as electroacupuncture [128]. Furthermore, specific compounds exert regulatory effects by directly inhibiting pro-ferroptotic enzymes (e.g., baicalein-mediated ALOX5 inhibition [29]) or modulating upstream signaling molecules (e.g., AMPK activation by metformin [129] or STAT1 inhibition by berberine [130]). Canonical ferroptosis inhibitors, such as Fer-1 [131]and liproxstatin-1 [132], are typically employed as positive controls to validate the involvement of ferroptosis via the direct scavenging of lipid peroxyl radicals.

### Iron Metabolism Regulation

This strategy primarily inhibits lipid peroxidation at its source by depleting the intracellular labile iron pool, thereby attenuating the Fenton reaction. Iron chelators, such as deferoxamine [132] and deferasirox [70], directly decrease the bioavailability of Fe^2+^ by sequestering free iron. Furthermore, specific natural products, such as isorhamnetin [62], exhibit dual functionality by simultaneously chelating iron and activating the Nrf2 pathway. Other compounds function through distinct molecular mechanisms; for example, liquiritin activates Prdx6 and upregulates FTH1 expression to increase ferritin-mediated iron storage [133], whereas arbutin reduces intracellular iron accumulation by modulating the Eno3-IRP1 axis [116].

### Lipid Metabolism Reprogramming

This strategy primarily targets PUFA synthesis and subsequent peroxidation. The inhibition of ACSL4 reduces the incorporation of PUFAs into membrane phospholipids, thereby limiting substrate availability for lipid peroxidation. Representative interventions include vitamin D [81] and the TCM formula Anchang decoction [134]. Furthermore, natural products such as 6-gingerol [17] and magnolin [29] attenuate the lipid peroxidation chain reaction by inhibiting lipoxygenases (e.g., ALOX5/15) or cyclooxygenase-2 (PTGS2). Vanillic acid acts via a distinct mechanism by upregulating SCD1 expression through the CA9/STIM1 pathway to promote MUFA synthesis [112], thereby exerting an anti-ferroptotic effect.

### Immune Cell Modulation

Recent evidence has indicated that immune cells, particularly macrophages and T cells, are pivotal in the pathogenesis of ferroptosis-mediated UC. For instance, β-caryophyllene inhibits macrophage ferroptosis and mitigates the subsequent release of pro-inflammatory cytokines by activating cannabinoid receptor type 2 (CB2R) [135]. Similarly, magnolin induces macrophage polarization toward the anti-inflammatory M2 phenotype [29]. Among more advanced therapeutic interventions, mineralized nanoinhibitors facilitate the targeted delivery of ferroptosis inhibitors while concurrently promoting M2 polarization via the CaSR/AKT/β-catenin signaling pathway [136]. Furthermore, mesenchymal stem cells (MSCs) and MSC-derived exosomes (e.g., ERC-exos [137] and hucMSC-Ex [138]) suppress ferroptosis in both immune and epithelial cells by delivering specific miRNAs (such as miR-129-5p, which targets ACSL4) or by upregulating the expression of protective molecules such as MUC-1.

### Gut Microbiota Modulation

The gut microbiota, which acts as a critical intermediary linking diet, host metabolism, and ferroptosis, has emerged as a crucial therapeutic target in UC. Various natural compounds (e.g., hesperetin [110], phlorizin [139], and protocatechuic acid [140]) in addition to sodium butyrate [54] can remodel the microbial composition by enriching beneficial taxa, such as *Lactobacillus*, Firmicutes, and *Prevotella*, while simultaneously suppressing opportunistic pathogens, including Proteobacteria, Erysipelotrichaceae, and *Clostridium*. This microbial shift enhances the production of protective metabolites, particularly SCFAs, which indirectly suppress ferroptosis in IECs. Specific interventions, such as the iron chelator deferasirox, function by simultaneously chelating iron and modulating the microbiota [70]. Additionally, targeted delivery systems such as probiotic-loaded microspheres prolong the colonic retention of probiotics, effectively attenuating localized ROS accumulation and iron deposition. Furthermore, microbe-derived ether lipids (e.g., plasmalogens [141]) have recently been identified as novel, direct inhibitors of ferroptosis.

### Multi‑Target/Integrated Regulation

Numerous multicomponent interventions, particularly TCM formulations (e.g., Shaoyao [142] and Gegen Qinlian [79] decoctions, as well as Kuijiening acupoint application [143]), have synergistic effects on the modulation of ferroptosis-related pathways. Their mechanisms of action are highly diverse and include the activation of the Nrf2/GPX4 axis, the modulation of iron metabolism, and the regulation of inflammatory signaling cascades, including those involving NF-κB and STAT3. Conversely, pathogenic bacteria such as *Fusobacterium nucleatum* can induce ferroptosis by downregulating GPX4 while upregulating ACSL4 and FTH1 [144], thereby compromising intestinal epithelial barrier integrity. This pathogen-driven mechanism provides a critical alternative perspective for understanding the pathogenesis of UC. In the future, targeted genetic interventions—such as overexpressing NEDD4L [145] or IGF2BP2 [146] to stabilize GPX4 expression or silencing the expression of pro-ferroptotic genes (e.g., *IRF7* [147] and *LCN2* [148])—represent promising avenues for precision therapy.

In summary, strategies for targeting ferroptosis in the treatment of UC involve the formation of a multitarget and multilayered regulatory landscape. These approaches include enhancing antioxidant defenses, intervening in iron and lipid metabolism, modulating immune cell function, and remodeling the gut microbiota. By acting upon distinct nodes within the ferroptotic cascade, they collectively reveal a complex network governing IEC death and offer multiple insights for potential combinatorial therapeutic regimens.

## Conclusions and Prospects

The identification of IEC ferroptosis broadens our understanding of UC pathogenesis, shifting the focus from purely immune-centric models to a highly integrated network of metabolic, nutritional, and redox dysregulation. Iron-dependent lipid peroxidation (PUFA–PL–OOH) serves not only as a secondary byproduct of inflammation but also as a primary driver of mucosal barrier dysfunction. By releasing DAMPs and activating the NLRP3 inflammasome, IEC ferroptosis promotes a self-amplifying cycle of chronic intestinal injury.

However, clinical translation faces substantial bottlenecks. Current strategies relying on broad-spectrum antioxidants, systemic iron chelators, or nonspecific lipid inhibitors pose significant off-target effects, as systemic interventions may disrupt the essential physiological functions of ROS and iron in antimicrobial defense (e.g., macrophage oxidative bursts) and energy metabolism. Therefore, future research must transition from phenomenological validation to achieving spatiotemporal precision in ferroptosis modulation. To bridge this translational gap, future investigations should prioritize the following frontiers: (1) Delineating spatiotemporal dynamics: studies must clarify whether transient ferroptosis acts as a protective mechanism to clear irreparably damaged IECs during acute injury, in contrast to its pathogenic role in chronic inflammation. Decoding this dichotomy is essential for defining optimal therapeutic windows. (2) Advancing precision delivery and biomarker stratification: to circumvent systemic toxicity, developing gut-targeted delivery systems (e.g., nanocarriers and hydrogels) is critical. Concurrently, identifying noninvasive biomarkers—such as distinct fecal lipidomic profiles—is vital for patient stratification and disease-stage-specific trials. (3) Decoding microenvironmental crosstalk: elucidating the bidirectional interactions among ferroptotic IECs, the enteric neuroimmune axis, and the gut metabolome is crucial. Specifically, investigating how microbial metabolites and neuropeptides metabolically and epigenetically affect IEC susceptibility to ferroptosis represents a promising therapeutic strategy.

In summary, mapping the ferroptotic regulatory network in UC has revealed critical therapeutic vulnerability. Integrating advanced multi-omics, targeted bioengineering, and patient-stratified trial designs to specifically modulate IEC ferroptosis has the potential to overcome the efficacy limits of current immunosuppressive therapies, paving the way for precision mucosal-healing strategies in IBD.

## Data Availability

Data availability is not applicable to this article as no new data were created or analyzed in this study.
